# Supplementary material for: Strong G-Protein-Mediated Inhibition of Sodium Channels
Source: Cell Rep. Author manuscript; Available in PMC 2018 Oct 26. (PMC6203318; doi:10.1016/j.celrep.2018.04.109)
Supplement: 1 [file NIHMS1508930-supplement-1.pdf]

Cell Reports, Volume *23*

Supplemental Information

Strong G-Protein-Mediated Inhibition of Sodium Channels

Glynis B. Mattheisen, Timur Tsintsadze, and Stephen M. Smith

SUPPLEMENTAL FIGURES AND LEGENDS

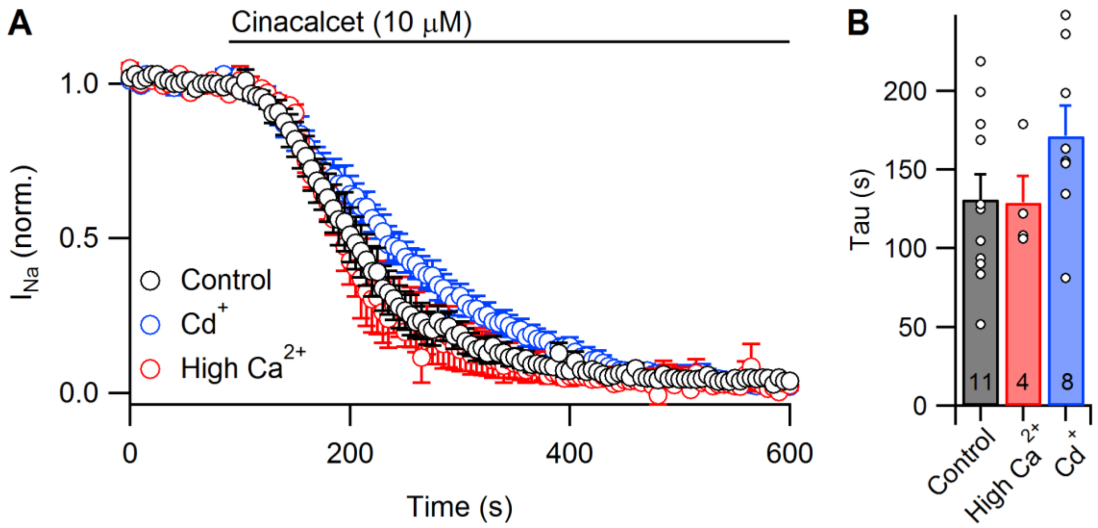

Figure S1. Cinacalcet inhibition is independent of extracellular calcium. Related to Figure 2. (A) Diary plot of VGSC current in cultured neocortical neurons during bath application of 10 µM cinacalcet in control condition (black; n =11), in the presence of 50 µM cadmium (Cd^{+}; n = 8; blue) to block VACCs, and in the presence of 10 mM extracellular calcium (High Ca^{2+}; n = 4; red). VGSC current was measured with 5 ms steps from -70 mV to 0 mV at 0.2 Hz. (B) Bar graph showing the time constant of the inhibition (Tau) by cinacalcet (10 µM) in control conditions (black), in the presence of 50 µM cadmium (Cd^{+}; blue), and in the presence of 10 mM extracellular calcium (High Ca^{2+}; red). Error bars show mean value ± SEM.

Table S1. GTPγS accelerated VGSC rundown compared with GTP and GDPβS. Related to Figure 3H.

**ANOVA table**	**SS**	**DF**	**MS**	**F (DFn, DFd)**	**P value**
**Interaction**	1.162	68	0.01710	$F(68, 1768) = 2.127$	$P < 0.0001$
**Time**	13.25	34	0.3898	$F(34, 1768) = 48.50$	$P < 0.0001$
**nucleotide**	5.993	2	2.997	$F(2, 52) = 3.708$	$P = 0.0312$
**Subjects (matching)**	42.03	52	0.8082	$F(52, 1768) = 100.6$	$P < 0.0001$
**Residual**	14.21	1768	0.008038		

SUPPLEMENTAL EXPERIMENTAL PROCEDURES

Resource Table

REAGENT or RESOURCE	SOURCE	IDENTIFIER
**Chemicals, Peptides, and Recombinant Proteins**		
Cinacalcet	Toronto Research Chemicals	Cat#C441803
Calindol	Sigma-Aldrich	CAS: 729610-18-8
NPS 2143	Tocris Bioscience	Cat#3626
Calhex	Sigma-Aldrich	CAS: 652973-93-8
**Experimental Models: Organisms/Strains**		
Mus musculus: C57/BL6J & 129S4: $^{nes\text{-}cre}CaSR^{\Delta flox/\Delta flox}$	Laboratory of Dr. Wenhan Chang	Chang, *et al.,* 2008
Mus musculus: C57BL/6J x 129X1: A^{W}/A^{W}	The Jackson Laboratory	RRID:MGI:5652742
