# Supplementary material for: Strong G-Protein-Mediated Inhibition of Sodium Channels
Source: Cell Rep. Author manuscript; Available in PMC 2018 Oct 26. (PMC6203318; doi:10.1016/j.celrep.2018.04.109)
Supplement: 2 [file NIHMS1508930-supplement-2.pdf]

Cell Reports

Article

Strong G-Protein-Mediated Inhibition of Sodium Channels

Graphical Abstract

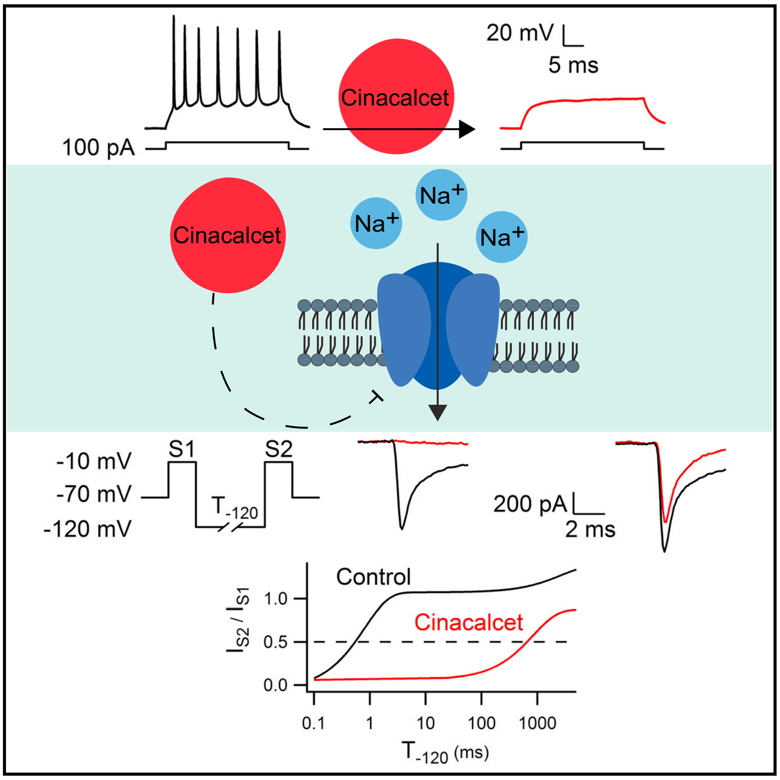

Highlights

- Calcium-sensing receptor modulators strongly inhibit VGSC currents in CNS neurons
- Pathway is ubiquitous in neocortical/hippocampal neurons and G-protein dependent
- G-protein-mediated inhibition of VGSC currents mediated by changes in VGSC gating
- This facilitates inactivation and profoundly slows hyperpolarization-mediated recovery

Authors

Glynis B. Mattheisen, Timur Tsintsadze, Stephen M. Smith

Correspondence

smisteph@ohsu.edu

In Brief

Mattheisen et al. demonstrate a G-protein-dependent pathway that strongly inhibits voltage-gated sodium channel currents in the vast majority of cortical neurons. The mechanism involves profound slowing of recovery from inactivation. The strong and widespread effects on voltage-gated sodium channels position this signaling pathway to have substantial influence on neuronal excitability.

Mattheisen et al., 2018, Cell Reports *23*, 2770–2781
May 29, 2018
https://doi.org/10.1016/j.celrep.2018.04.109

CellPress

OPEN ACCESS
CellPress

Cell Reports
Article

Strong G-Protein-Mediated Inhibition of Sodium Channels

Glynis B. Mattheisen,[1,2] Timur Tsintsadze,[1,2] and Stephen M. Smith[1,2,3,*]
[1]Department of Medicine, Division of Pulmonary & Critical Care Medicine, Oregon Health & Science University, Portland, OR 97239, USA
[2]Section of Pulmonary & Critical Care Medicine, VA Portland Health Care System, Portland, OR 97239, USA
[3]Lead Contact
*Correspondence: smisteph@ohsu.edu
https://doi.org/10.1016/j.celrep.2018.04.109

SUMMARY

Voltage-gated sodium channels (VGSCs) are strategically positioned to mediate neuronal plasticity because of their influence on action potential waveform. VGSC function may be strongly inhibited by local anesthetic and antiepileptic drugs and modestly modulated via second messenger pathways. Here, we report that the allosteric modulators of the calcium-sensing receptor (CaSR) cinacalcet, calindol, calhex, and NPS 2143 completely inhibit VGSC current in the vast majority of cultured mouse neocortical neurons. This form of VGSC current block persisted in CaSR-deficient neurons, indicating a CaSR-independent mechanism. Cinacalcet-mediated blockade of VGSCs was prevented by the guanosine diphosphate (GDP) analog GDPβs, indicating that G-proteins mediated this effect. Cinacalcet inhibited VGSCs by increasing channel inactivation, and block was reversed by prolonged hyperpolarization. Strong cinacalcet inhibition of VGSC currents was also present in acutely isolated mouse cortical neurons. These data identify a dynamic signaling pathway by which G-proteins regulate VGSC current to indirectly modulate central neuronal excitability.

INTRODUCTION

Voltage-gated sodium channels (VGSCs) drive the action potential and are integral to neuronal function. However, the picture of the action potential as a digital all-or-none signal has evolved with the identification of persistent and regenerative types of VGSCs that produce variation in the action potential shape between neuronal types (Huang and Trussell, 2008; Raman and Bean, 1997). Additional variation in action potential waveform arises from several types of endogenous VGSC regulation, including altered inactivation by β subunit interactions (Aman et al., 2009), regional variation in sodium channel density (Leão et al., 2005), and increased persistent VGSC current arising from the inherited β subunit mutations that influence excitability (Kaplan et al., 2016). These indirect mechanisms regulate VGSC signaling and thereby account for action potential variation between neurons. However, such effects are stable over short periods of time. In contrast, local anesthetics and antiepileptic drugs target VGSCs and rapidly modulate action potentials by stabilizing channel inactivation (Kuo and Bean, 1994; Zeng et al., 2016). In addition, dynamic modulation of VGSCs via calmodulin (Pitt and Lee, 2016) and G-protein-coupled receptors (GPCRs) (Carr et al., 2003) has also been proposed to contribute to neuronal plasticity.

The calcium-sensing receptor (CaSR) is a GPCR expressed in many tissues, including those of the nervous system (Leach et al., 2015). In the cerebral cortex, CaSRs are expressed at nerve terminals (Chen et al., 2010), where they modulate evoked and spontaneous synaptic transmission (Phillips et al., 2008; Smith et al., 2012). Here, we report that allosteric CaSR modulators (ACMs) reduced GABAergic transmission between neocortical neurons and that this was attributable to block of VGSCs. Further examination showed that both allosteric agonists and antagonists of the CaSR completely inhibited VGSC current. This block of VGSC current was independent of the CaSR but required G-protein activation. The CaSR allosteric agonist cinacalcet inhibited VGSC current by negatively shifting steady-state inactivation of the channels. This cinacalcet-induced inhibition was reversed by prolonged hyperpolarization. The VGSC inhibition appeared independent of class C GPCRs and occurred through a protein kinase A (PKA)-independent and protein kinase C (PKC)-independent pathway. These data describe an important mechanism for modulating neuronal excitability in the cortex.

RESULTS

Allosteric CaSR Agonists Reduce VGSC Current

Direct CaSR agonists produced a graded inhibition of synaptic transmission in neocortical neurons (Phillips et al., 2008), leading us to hypothesize that cinacalcet, an allosteric agonist of the CaSR, would have the same effect. We evoked inhibitory postsynaptic currents (IPSCs) by stimulating presynaptic neurons with a theta electrode (Figure 1A). Application of cinacalcet (10 μM) almost completely eliminated IPSCs within 100–200 s (Figure 1B; 96% ± 1% [mean ± SEM] block in eight recordings). In this neuron, voltage clamped at −70 mV, IPSC amplitude ranged from 70–200 pA, and quantal size was 30–40 pA. The initial effect of cinacalcet appeared to be all or none, and we hypothesized that this was due to block of the presynaptic action potential, leading to the coordinated block of multiple presynaptic GABA release sites. Consistent with this finding, somatic action potentials were blocked by cinacalcet (Figure 1C). We next tested if cinacalcet modulated VGSC currents elicited in

This is an open access article under the CC BY license (http://creativecommons.org/licenses/by/4.0/).

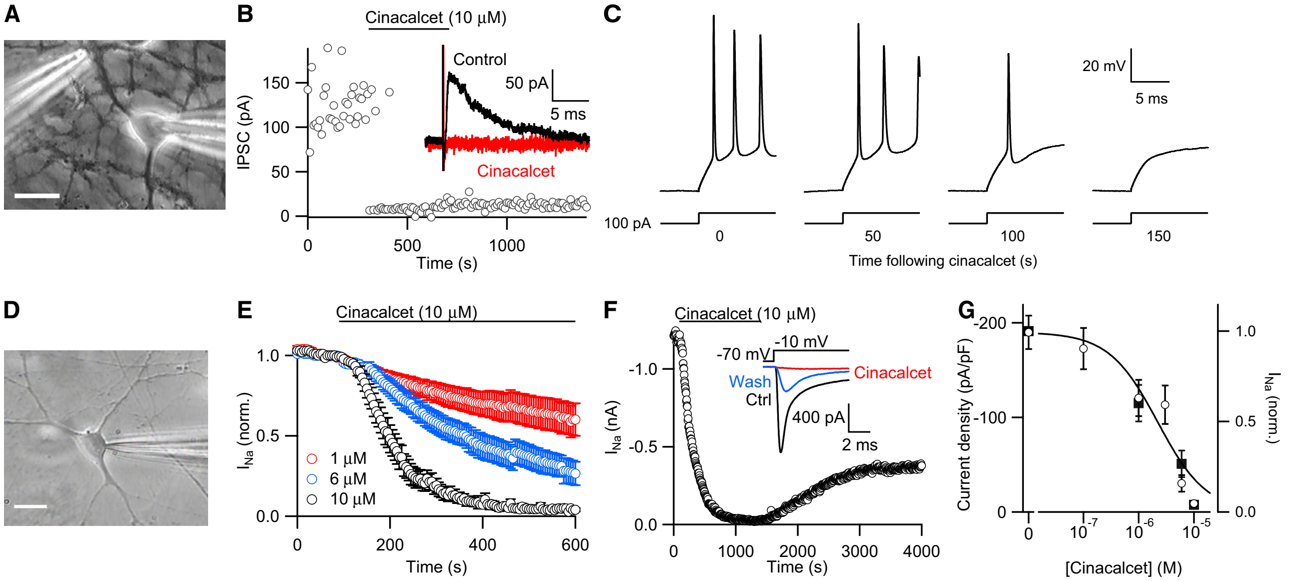

Figure 1. Inhibition of VGSC Current by CaSR Allosteric Agonist Cinacalcet

(A) Image of whole-cell voltage-clamp recording from a cultured neocortical neuron with theta electrode used to evoke IPSCs. Scale bar indicates 10 μm.

(B) Diary plot showing IPSC amplitude reduced by 10 μM cinacalcet (application indicated by horizontal bar in this and later figures). Inset: representative IPSCs in vehicle control (black) and after steady-state effect of cinacalcet (red).

(C) Current-clamp recordings showing action of cinacalcet on response to 100 pA current injections.

(D) Image of whole-cell recording from a cultured neocortical neuron. Scale bar indicates 10 μm.

(E) Diary plot of average normalized peak VGSC current elicited by a 30 ms test pulse to −10 mV from a holding potential of −70 mV every 5 s during perfusion of 10 μM (n = 11), 6 μM (n = 9), or 1 μM cinacalcet (n = 9).

(F) Exemplar diary plot of peak VGSC current elicited as in (E) following application of 10 μM cinacalcet. Inset: representative VGSC currents in control conditions (ctrl, black), at maximal block (red), and at maximal recovery (blue).

(G) Concentration-effect relationship for cinacalcet on VGSC currents. Left axis indicates current density following incubation in cinacalcet for 50–70 min (open circles). VGSC amplitude was measured immediately following whole-cell formation (same protocol as E) and normalized to measured cell capacitance (n ≥ 10 for each group). Data fit with Hill equation with IC_{50} = 3.5 ± 1 μM cinacalcet and Hill coefficient = 0.98. Right axis: normalized VGSC current from (E), 510 s following the application of cinacalcet (solid squares). Data are plotted as mean ± SEM in this and later figures.

voltage-clamped neurons (30 ms step from −70 to −10 mV every 5 s; Figures 1D–1F). Tetrodotoxin (TTX; 1 μM) reversibly reduced the rapidly activating and inactivating inward current (peak < 1 ms) by 98% ± 1% (n = 6, data not shown), confirming that these conditions isolated the VGSC current. Application of cinacalcet (10 μM) strongly inhibited the peak VGSC current by 98% ± 1% (n = 11), and the kinetics of block were described by a single exponential (T = 61 ± 8 s) after a delay of 73 ± 9 s (Figure 1E). VGSC current inhibition by cinacalcet was concentration dependent (Figure 1E) but reversed slowly and incompletely with the −70 mV holding potential (Figure 1F; but see Figure 6).

The concentration-effect relationship for cinacalcet was determined by measuring the VGSC current immediately following whole-cell formation after incubation (50–70 min) in the drug. This approach was used because cinacalcet was effective in all neocortical neurons (>300 recordings) and because at lower concentrations, the slower rate of block and current rundown could have confounded measurement of the half maximal inhibitory concentration (IC_{50}). VGSC current density (pA/pF) was inversely related to cinacalcet concentration in neocortical neurons after 7–9 days in culture (IC_{50} = 2.2 ± 0.6 μM; Figure 1G). This was in agreement with the degree of block measured when we examined the time course of inhibition with 1–10 μM cinacalcet (Figures 1E and 1G, solid squares). These data show that cinacalcet strongly inhibits VGSCs in a concentration-dependent manner in neocortical neurons.

ACMs Inhibit VGSCs by a CaSR-Independent Pathway

We hypothesized that cinacalcet inhibited VGSCs via the target CaSR and tested this idea first by examining if other CaSR modulators inhibited VGSC current. Calindol (5 μM), another CaSR allosteric agonist, strongly inhibited peak VGSC current (Figures 2A and 2D; 97% ± 1% steady-state inhibition, n = 5) elicited as above (see Figure 1E). Next, we tested if the CaSR was the target of these drugs by examining if VGSC current was insensitive to cinacalcet in neurons from CaSR null mutants ($Casr^{-/-}$) using the same protocol (Chang et al., 2008). Surprisingly, VGSC currents from $Casr^{-/-}$ and wild-type neurons were equally sensitive to cinacalcet (Figures 2B and 2D; 100% ± 1%, n = 12, p = 0.2). The time constant of the inhibition and latency of the effect of cinacalcet were also unchanged. Furthermore, direct stimulation of the CaSR by increasing the external calcium concentration to 10 mM, did not change the kinetics of VGSC block by 10 μM cinacalcet (Figure S1). These data indicated that cinacalcet-induced VGSC current inhibition is independent of the CaSR.

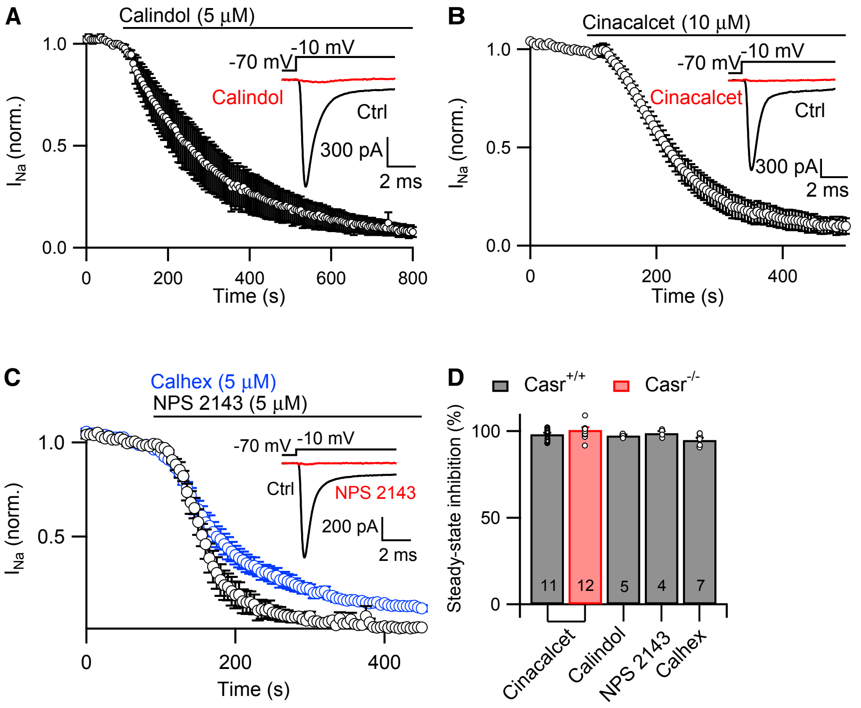

Figure 2. CaSR ACMs Inhibit VGSC Current in Wild-Type ($Casr^{+/+}$) and CaSR-Null ($Casr^{-/-}$) Mutants

(A) Diary plot of average normalized peak VGSC current elicited as in Figure 1E during perfusion of 5 μM calindol (n = 5). Inset: representative traces show VGSC current in control (ctrl, black) conditions and after steady-state block by calindol (red).

(B) Diary plot of average normalized VGSC current (elicited as in Figure 1E) during perfusion of 10 μM cinacalcet recorded in $Casr^{-/-}$ neocortical neurons (n = 12). Inset: representative traces show VGSC current in control conditions (ctrl, black) and after steady-state inhibition by cinacalcet (red).

(C) Diary plot of average normalized VGSC current (elicited as in Figure 1E) during bath perfusion of 5 μM calhex (blue, n = 7) or 5 μM NPS 2143 (black, n = 4) recorded in $Casr^{+/+}$ neocortical neurons. Inset: representative traces show VGSC current in control conditions (ctrl, black) and after steady-state inhibition by NPS 2143 (red).

(D) Bar graph summarizing the effects of 10 μM cinacalcet, 5 μM calindol, 5 μM calhex, or 5 μM NPS 2143 on VGSC current in $Casr^{+/+}$ (black) and $Casr^{-/-}$ (red) neocortical neurons. Number of recordings in each condition indicated at the foot of each bar in this and later histograms.

Error bars represent mean ± SEM.

Upregulation of other similar compensatory proteins could explain why $Casr^{-/-}$ neurons responded to CaSR agonists. Thus, we tested the effect of the allosteric CaSR antagonists NPS 2143 and calhex on VGSC currents (elicited as in Figure 1E). NPS 2143 (5 μM) and calhex (5 μM) strongly blocked VGSC currents (Figures 2C and 2D; 99% ± 1% [n = 4] and 95% ± 1% [n = 7], respectively). Both agents also inhibited VGSC currents in $Casr^{-/-}$ neurons (data not shown). These data show that ACMs inhibit VGSC currents in wild-type and $Casr^{-/-}$, strongly indicating that these effects are not mediated by the CaSR.

G-Protein-Mediated Changes to VGSC Current

To determine if the cinacalcet-induced block of VGSC current relied on G-protein signaling, we tested the effect of the guanosine diphosphate (GDP) analog GDPβS on the cinacalcet-induced response. GDPβS inhibits G-protein cycling by competitively inhibiting the binding of guanosine triphosphate (GTP) to G-proteins (Eckstein et al., 1979; Suh et al., 2004). Cinacalcet (10 μM) inhibited VGSC current by 90% ± 3% (n = 10), measured 250 s after onset of application, with 0.3 mM GTP in the recording pipette solution. In contrast, cinacalcet reduced VGSC current by only 8% ± 3% (n = 12, $p = 7 \times 10^{-15}$) at the same time point with 2 mM GDPβS in the pipette solution (Figures 3A and 3B). GDPβS also reduced calindol-induced inhibition to 29% ± 8% (n = 6) at 250 s compared with 66% ± 11% (n = 5, p = 0.02) in the control conditions (Figure 3B). The block of VGSCs by CaSR allosteric antagonists NPS 2143 and calhex was also G-protein mediated. NPS 2143-induced inhibition was reduced from 96% ± 1% (n = 4) 250 s following NPS 2143 exposure to 5% ± 13% at the same time point in the presence of GDPβS (2 mM) (n = 7, p = 0.0005; Figures 3C and 3D). Calhex-induced inhibition was reduced from 82% ± 4% (n = 7) at 250 s to 33% ± 10% at the same time point in the presence of GDPβS (n = 7, p = 0.0008; Figure 3D).

We asked three questions to address the possibility that the four ACMs inhibited VGSCs via GDPβS-sensitive pathways that did not involve G-proteins. First, was GDPβS chemically inactivating the ACMs after they reached the intracellular compartment? The subsequent action of cinacalcet on VGSC currents was unaffected following preincubation with GDPβS (2 mM for 30 m at room temperature), indicating that GDPβS was not simply inactivating the ACMs (data not shown). Second, was GDPβS interfering with ACM inhibition of VGSC currents because of an action of the non-hydrolyzable part of the molecule? Like GDPβS, ADPβS is non-hydrolyzable because of an oxygen-to-sulfur switch at the terminal phosphate (Cusack and Hourani, 1981) but extremely unlikely to bind to the tight nucleotide pocket of Gα (Lambright et al., 1994; Oldham and Hamm, 2008). Unlike with GDPβS, the ADPβS (2 mM) in the pipette did not slow or reduce the inhibition of VGSC current by cinacalcet (n = 8) compared with our control condition in recordings with 300 μM GTP (n = 12; Figures 3E and 3F). Third, did GDPβS alter VGSC resistance to direct blockers and thus reduce the effectiveness of ACMs? To address this question, we tested if GDPβS affected the actions of other VGSC blockers (Rogawski et al., 2016). VGSC currents were reduced by 37% ± 5% and 46% ± 7% by the application of carbamazepine (Figure 3G; 100 μM, n = 7) and phenytoin (100 μM, n = 9) respectively. This effect was unchanged by GDPβS (Figure 3G; 32% ± 5% for carbamazepine, n = 9; 39% ± 6% for phenytoin, n = 9), indicating that GDPβS was not simply increasing VGSC resistance to

OPEN
ACCESS
CellPress

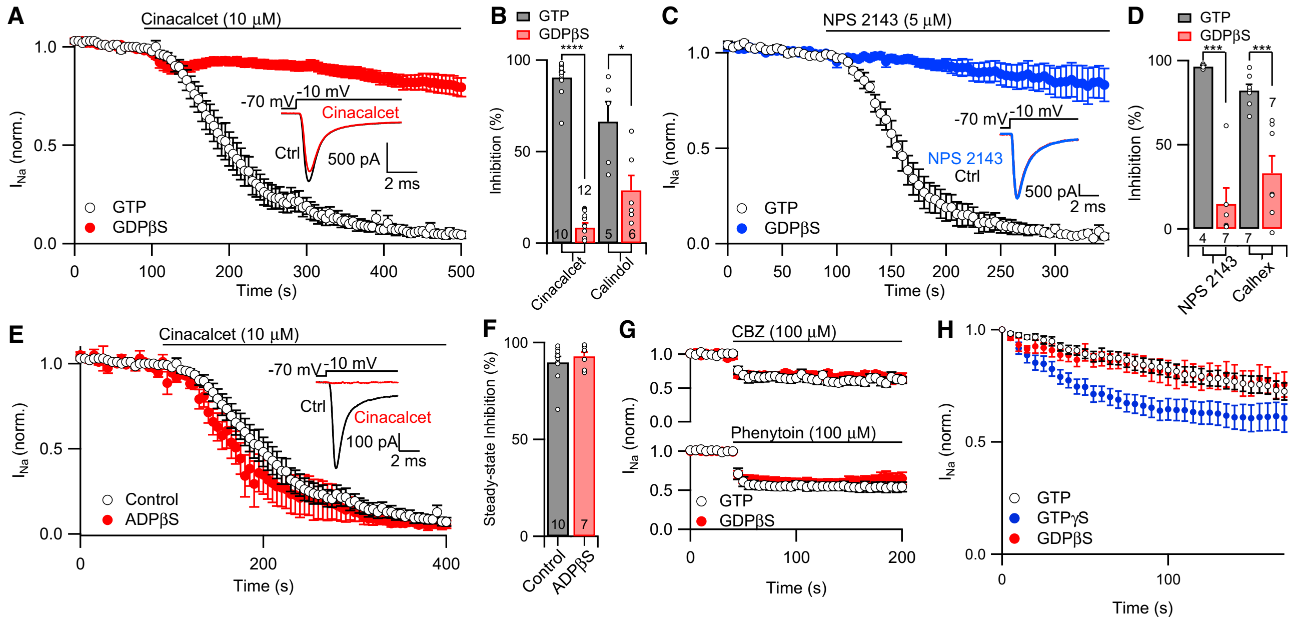

Figure 3. Allosteric CaSR Modulator Block of VGSC Current Is GTP Dependent

(A) Plot of average normalized VGSC current (elicited as for Figure 1E) during perfusion of 10 μM cinacalcet in control conditions (black, n = 10) or with 2 mM GDPβS in pipette solution (red, n = 12). Inset: representative traces show VGSC current baseline in the presence of 2 mM GDPβS prior to (ctrl, black) and at 250 s after the application of cinacalcet (red).

(B) Bar graph summarizing the effects of 10 μM cinacalcet and 5 μM calindol on VGSC current in control conditions (black) and in recordings with 2 mM GDPβS (red) 250 s following drug exposure.

(C) Plot of average normalized VGSC current (elicited as in Figure 1E) during perfusion of 5 μM NPS 2143 recorded in control conditions (black, n = 4) and with 2 mM GDPβS (blue, n = 7) in recording solution. Inset: representative traces show VGSC current baseline in the presence of 2 mM GDPβS prior to (ctrl, black) and at the time point 250 s after the application of NPS 2143 (blue).

(D) Bar graph summarizing the effects of 5 μM NPS 2143 and 5 μM calhex on VGSC current in control conditions (black) and with 2 mM GDPβS (red) after 250 s of drug application.

(E) Plot of average normalized VGSC current (elicited as in Figure 1E) during perfusion of 10 μM cinacalcet recorded in control conditions (black, n = 10) and with 2 mM ADPβS (red, n = 7) in the recording solution. Inset: representative traces show VGSC current baseline in the presence of 2 mM ADPβS prior to (ctrl, black) and at the time point 250 s after the application of cinacalcet (red).

(F) Bar graph summarizing the effects of 10 μM cinacalcet on VGSC current in control conditions (black) and in recordings with 2 mM ADPβS (red) 250 s following drug exposure.

(G) Diary plot of average normalized VGSC current (elicited as in Figure 1E) during perfusion of 100 μM carbamazepine (top) or 100 μM phenytoin (bottom) recorded in control conditions (black; phenytoin, n = 9, carbamazepine, n = 7) and with 2 mM GDPβS (red; phenytoin, n = 9; carbamazepine, n = 9) in pipette solution.

(H) Plot of average normalized VGSC current (elicited as for Figure 1E) with 0.3 mM GTP (black), 2 mM GDPβS (red), or 500 μM GTPγS (blue) in the pipette solution.

Error bars represent ± SEM.

*$p < 0.05$, ***$p < 0.001$, and ****$p < 0.0001$.

direct inhibitors. These experiments are consistent with GDPβS inhibiting ACM-mediated inhibition of VGSC currents via a GTP-dependent mechanism.

Basal activity of G-proteins has been reported in many systems arising from constitutive activity or low basal activation of the GPCR (Seifert and Wenzel-Seifert, 2002). We hypothesized that GTPγS may accelerate rundown of VGSC current in the absence of ACMs if there was basal activity of this signaling pathway. VGSC currents were activated with 30 ms steps from −70 to −10 mV at 0.2 Hz, and recordings were made with GTP (0.3 mM), GDPβS (2 mM), or GTPγS (500 μM) in the pipette solution (Figure 3H). GTPγS accelerated VGSC rundown compared with GTP and GDPβS (Figure 3H; two-way ANOVA with repeated measures [RM], interaction $F[68, 1{,}768] = 2.13$, $p < 0.0001$). With GTP and GDPβS in the pipette, VGSC currents decreased by 36% ± 4% (n = 20) and 34% ± 6% (n = 24) during the first 5 min of recording, whereas the same decrease occurred in 95 s in the presence of GTPγS (n = 12). These data indicate that ACM-induced inhibition of VGSCs is independent of the CaSR but dependent on G-proteins.

Molecular Targets for G-Protein-Mediated VGSC Inhibition

To identify potential targets for cinacalcet, we tested if its action was affected by antagonists to GPCRs structurally similar to CaSR (Urwyler, 2011). VGSC current was elicited with voltage steps to −10 mV, and neurons were perfused with an mGluR1 or mGluR5 blocker (competitive antagonist or negative allosteric modulator) for a minimum of 120 s before the application of cinacalcet (6 μM) (Figure 4A). Perfusion of the blockers continued during the application of cinacalcet. As above, VGSC currents were elicited with 30 ms steps from −70 to −10 mV at 0.2 Hz. None

OPEN
ACCESS
CellPress

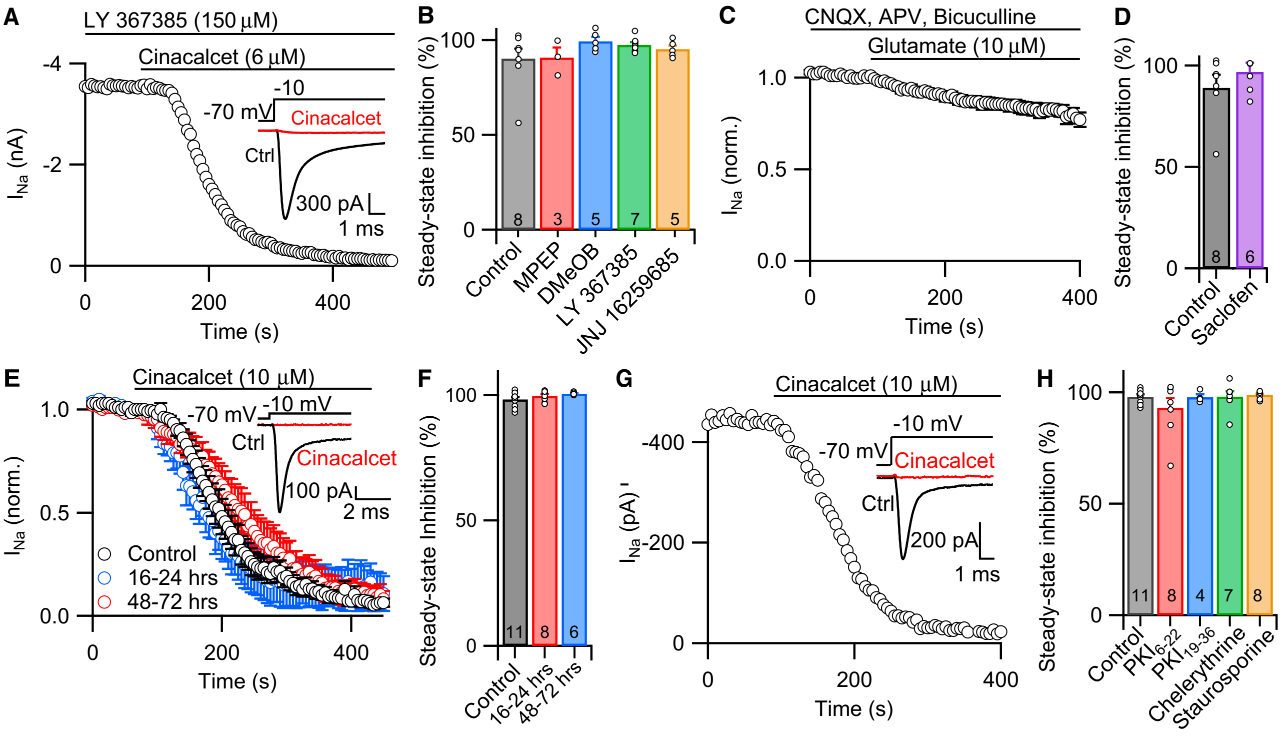

Figure 4. Cinacalcet-Induced Inhibition of VGSC Current Is Not Mediated by mGluR1, mGluR5, or GABA$_B$ Receptors nor Does It Require Activation of PKA or PKC

(A) Exemplar plot of peak VGSC current (elicited as in Figure 1E) during perfusion of 6 μM cinacalcet. mGluR1 antagonist LY 367385 (150 μM) was applied for a minimum of 160 s prior to and during the perfusion of cinacalcet. Inset: representative traces show VGSC current in control (ctrl, black) conditions and after steady-state block by cinacalcet (red).

(B) Bar graph summarizing the effects of 6 μM cinacalcet on VGSC current in the presence of mGluR1 or mGluR5 antagonists and negative allosteric modulators (30 μM MPEP, 50 μM DMeOB, 150 μM LY, and 500 nM JNJ) perfused a minimum of 2 min prior to and during cinacalcet perfusion.

(C) Diary plot of average normalized VGSC current (elicited as in Figure 1E) during perfusion of 10 μM glutamate (n = 9) in the presence of ionotropic glutamate receptor antagonists CNQX (10 μM), APV (50 μM), and bicuculline (10 μM).

(D) Bar graph summarizing the effects of 6 μM cinacalcet on VGSC current in the presence of GABA$_B$ receptor antagonist saclofen (500 μM) perfused a minimum of 2 min prior to and during cinacalcet application.

(E) Plot of average normalized VGSC current (elicited as in Figure 1E) during perfusion of 10 μM cinacalcet after 16–24 hr (n = 8) or 48–72 hr (n = 6) incubation in 200 ng/mL PTx versus control condition (n = 11). Inset: representative traces show VGSC current in control (ctrl, black) conditions and after steady-state block by cinacalcet (red) with 48–72 hr incubation in PTx.

(F) Bar graph summarizing the effects of 10 μM cinacalcet on VGSC current after 16–24 or 48–72 hr incubation with 200 ng/mL PTx.

(G) Exemplar plot of peak VGSC current (elicited as in Figure 1E) during perfusion of 10 μM cinacalcet in a recording with 5 μM PKC inhibitor PKI$_{19–36}$ in the recording pipette. Inset: representative traces show VGSC current in control (ctrl, black) conditions and after steady-state block by cinacalcet (red).

(H) Bar graph summarizing the effects of 10 μM cinacalcet on VGSC current with 20 μM PKI$_{6–22}$, 5 μM PKI$_{19–36}$, 10 μM chelerythrine chloride, or 100 nM staurosporine in the recording solution.

Error bars represent ± SEM.

of the mGluR1 and mGluR5 blockers tested (30 μM 2-methyl-6-[phenylethynyl]-pyridine [MPEP], 50 μM 3-methoxybenzaldehyde [(3-methoxyphenyl)methylene]hydrazone [DMeOB], 150 μM LY 367385, or 500 nM JNJ 16259685) slowed or reduced the cinacalcet-induced inhibition (Figure 4B). In addition, mGluR1 and mGluR5 agonists (*RS*)-2-chloro-5-hydroxyphenylglycine (CHPG; 100 μM) and (*S*)-3,5-dihydroxyphenylglycine (DHPG; 100 μM) did not inhibit VGSC currents, indicating that cinacalcet was not activating these receptors (data not shown). Similarly, application of glutamate (10 μM; applied in the presence of ionotropic glutamate receptors antagonists 6-cyano-7-nitroquinoxaline-2,3-dione [CNQX] [10 μM] and DL-2-amino-5-phosphonopentanoic acid [APV; 50 μM]) and the GABA$_A$ receptor antagonist bicuculline (10 μM) did not affect VGSC current (Figure 4C; n = 9). Next, we tested if cinacalcet acted through class C GPCRs GABA$_B$ receptors by applying cinacalcet in the presence of GABA$_B$ receptor antagonist saclofen. Reducing GABA$_B$ receptor activity with saclofen (500 μM) did not alter the cinacalcet-induced response (Figure 4D; n = 6). Furthermore, stimulation of GABA$_B$ receptors with baclofen (10 μM) did not significantly reduce VGSC current in a manner similar to that observed with the application of cinacalcet (data not shown). These data indicate that cinacalcet does not inhibit VGSCs through the activation of metabotropic glutamate receptors or GABA$_B$ receptors.

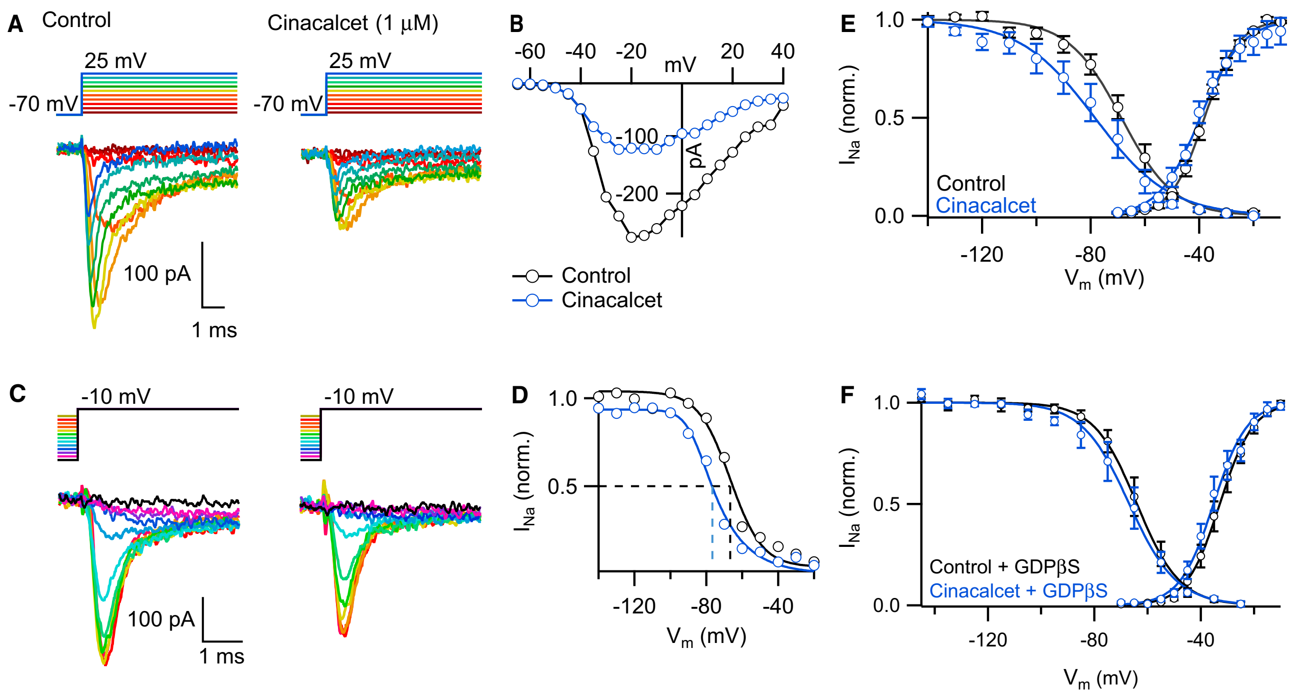

Figure 5. Cinacalcet Negatively Shifts Steady-State Inactivation of VGSCs in a G-Protein-Dependent Manner

(A) Representative traces from a protocol used to study the voltage dependence of activation wherein depolarizing steps are made from −70 mV holding potential to +40 mV at 5 mV intervals in control conditions (left) and at ~50% inhibition by 1 μM cinacalcet in the same cell (right).

(B) Single-cell current-voltage relationship in control conditions (black) and at ~50% inhibition by cinacalcet (blue) fit to the Boltzmann equation.

(C) Representative traces from a protocol used to study the voltage dependence of channel inactivation wherein test pulse to −10 mV are made following a 500 ms prepulse between −120 and −20 mV at 10 mV intervals in control conditions (left) and at ~50% inhibition by 1 μM cinacalcet in the same cell (right).

(D) Single-cell inactivation curves in control conditions (black) and at ~50% inhibition by cinacalcet (blue) fit to the Boltzmann equation and normalized to control data.

(E) Average activation and inactivation curves in control conditions (black) and at ~50% inhibition by cinacalcet (blue). The lines are fit to the Boltzmann equation (activation: $V_{0.5}$ control = −33 ± 1 mV, $V_{0.5}$ cinacalcet = −36 ± 1 mV, n = 11, p = 3 × 10^{-05}, paired t test; inactivation: $V_{0.5}$ control = −69 ± 3 mV, $V_{0.5}$ cinacalcet = −81 ± 5 mV, n = 15, p = 0.002, paired t test).

(F) Average activation and inactivation curves in control conditions (black) and at a time point at which ~50% inhibition by cinacalcet would be expected (blue) in recordings with 2 mM GDPβS. The lines are best fit to the Boltzmann equation (activation: $V_{0.5}$ control = −28 ± 2 mV, $V_{0.5}$ cinacalcet = −30 ± 2 mV, n = 12, p = 0.08, paired t test; inactivation: $V_{0.5}$ control = −59 ± 2 mV, $V_{0.5}$ cinacalcet = −63 ± 3 mV, n = 9, p = 0.054, paired t test).

Error bars represent ± SEM.

GPCRs can be coupled to a range of different G-protein complexes, the primary families being $G_{i/o}$, G_q, G_s, and G_{12} (Neves et al., 2002). Pertussis toxin (PTx) is a specific inhibitor of $G_{i/o}$ signaling (Ui, 1984). Preincubation with PTX (200 ng/mL) for either 16–24 hr (n = 8) or 48–72 hr (n = 6) did not alter the cinacalcet-induced inhibition of VGSC currents, indicating that the pathway was mediated by G-proteins other than $G_{i/o}$ (Figures 4F and 4G).

G-protein-activated phosphorylation of VGSCs by PKA and PKC reduces VGSC current by 20%−40% (Cantrell et al., 1999; Carlier et al., 2006; Carr et al., 2002). To test if these kinases mediate the cinacalcet-induced reduction in VGSC current, we performed whole-cell recordings with PKA- or PKC-specific blockers in the pipette solution (Figures 4G and 4H). The cinacalcet effects on steady-state inhibition, latency of action, and rate of inhibition of VGSC currents were unaffected by the PKA inhibitor $PKI_{6–22}$ (20 μM; Figure 4F; n = 8). Additionally, PKC inhibitors $PKI_{19–36}$ (20 μM; n = 4) and chelerythrine chloride (10 μM; n = 7) did not affect the action of cinacalcet on VGSC currents (Figure 4H). Furthermore, the broad-spectrum kinase inhibitor staurosporine (100 nM; n = 8) was also ineffective in the pipette solution (Figure 4H). Although the data do not rule out the involvement of staurosporine-resistant kinases, they indicate that cinacalcet-induced inhibition occurs in a PKA- and PKC-independent manner.

Cinacalcet Promotes Inactivation of VGSC Current

To determine how cinacalcet inhibited VGSC current, we evaluated the effect of cinacalcet on VGSC gating properties. Gating properties were tested in neocortical neurons with shorter processes (24–48 hr in culture to reduce space clamp errors). Activation was studied by eliciting VGSC currents with a series of 10 ms voltage steps from −70 mV to between −65 and +40 mV in 5 mV increments at 0.2 Hz (Figure 5A). Steady-state inactivation was then studied by activating VGSC currents with a 20 ms test pulse to −10 mV preceded by a 500 ms conditioning step to between −140 and −20 mV in 10 mV increments (Figure 5C). Cinacalcet (1 μM) was then applied until the VGSC

OPEN ACCESS
CellPress

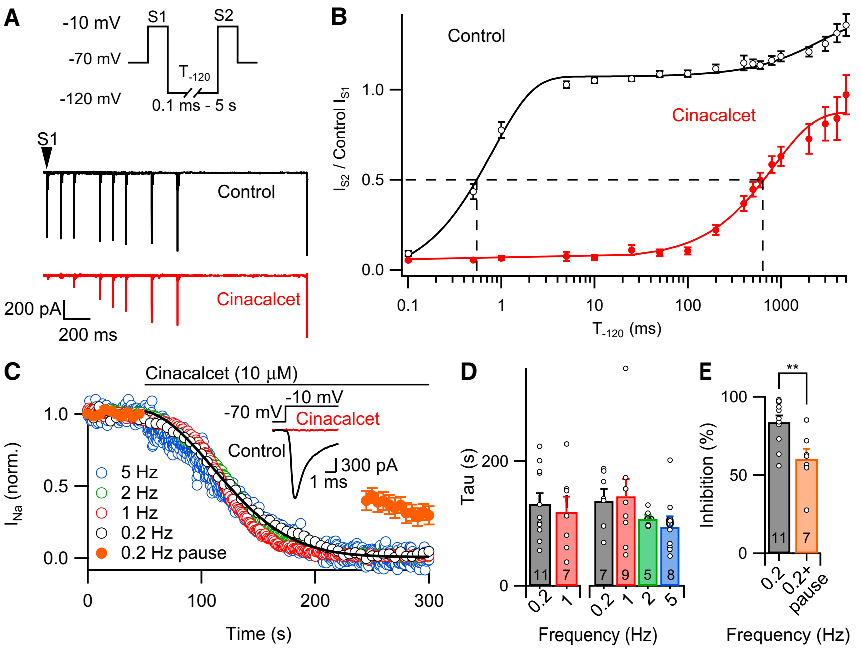

Figure 6. Cinacalcet Block Is Use Dependent and Recovers Following Hyperpolarization

(A) Representative traces from a double pulse protocol (S1 and S2) used to elicit VGSC currents in control (top, black) or after complete block by 10 µM cinacalcet (bottom, red). Test pulses S1 and S2 are 10 ms in length and separated by a variable-length recovery period at −120 mV.

(B) Graph showing double-exponential increase in VGSC current amplitude with increased time at −120 mV in control conditions (black) (T1 = 0.807 ± 0.055 ms, T2 = 2,583 ± 983 ms; n = 13) and single-exponential recovery of VGSC current after full inhibition with 10 µM cinacalcet (red) with increased period at −120 mV (T = 841 ± 72 ms; n = 10).

(C) Diary plot of normalized VGSC current elicited by a 5 ms test pulse from −70 to −10 mV at 0.2 Hz (black, open), 1 Hz (red, open), 2 Hz (green, open), 5 Hz (blue, open), or 0.2 Hz with pause (average of 7; orange, solid) during perfusion of 10 µM cinacalcet. Black line shows fit of 2 Hz using equation 1. Inset: representative traces show VGSC current in control conditions (ctrl, black) and at steady-state inhibition by cinacalcet (red) from the 2 Hz recording exemplar shown.

(D) Bar graph summarizing the effect of stimulation frequency on the time constant of cinacalcet inhibition of VGSC current by 10 µM cinacalcet. Currents elicited with 30 ms (left) or 5 ms (right) steps to −10 mV.

(E) Bar graph comparing the inhibition of VGSC current 200 s after cinacalcet application following sustained activation at 0.2 Hz (gray, n = 11) and following a 200 s pause in channel activation (red, n = 7). **$p < 0.01$.

Error bars represent ± SEM.

current had decreased by ~50% and VGSC current activation and inactivation reexamined. In the exemplar, cinacalcet reduced the peak VGSC currents by ~50% at voltages above −40 mV (Figure 5B). However, strong hyperpolarization reversed the inhibition of the VGSC current to only 10% (Figure 5D), consistent with cinacalcet promoting VGSC inactivation. Average conductance-voltage plots, derived from the current-voltage curves, were normalized to facilitate comparison of half-activation voltages ($V_{0.5}$). The change in $V_{0.5}$ ($\Delta V_{0.5}$) for the steady-state inactivation was strongly shifted (−11 ± 3 mV) by the application of cinacalcet; the average $V_{0.5}$ values for control and cinacalcet were −69 ± 3 and −81 ± 5 mV, respectively (Figure 5E; n = 11; p = 0.002). A smaller $\Delta V_{0.5}$ was seen for activation (Figure 5E; −33 ± 1 and −36 ± 1 mV in control and cinacalcet, respectively, n = 15; $p = 3 \times 10^{-5}$). The shift in gating confirms that cinacalcet promotes the inactivated state, thereby reducing the amplitude of the VGSC current. GDPβS also blocked the hyperpolarizing shift in steady-state inactivation of VGSCs (Figure 5F; $\Delta V_{0.5}$ = −3 ± 1 mV; n = 9; p > 0.05), consistent with the proposal that cinacalcet inhibits VGSCs by a G-protein-mediated mechanism that stabilizes the inactivated state.

Because strong hyperpolarization (−140 mV for 500 ms) only partially reversed the inactivation by cinacalcet, we tested if a greater fraction of inhibition was reversible with longer hyperpolarizing pulses (Jo and Bean, 2011; Karoly et al., 2010). A double-pulse protocol (S1 and S2, each −10 mV, 10 ms) was used to elicit VGSC currents (I_{S1} and I_{S2}) in control or after complete block by cinacalcet (10 µM; Figures 6A and 6B). I_{S2} was fully recovered within <10 ms in control experiments. After full block by cinacalcet, I_{S2} recovered to 98% ± 3% of I_{S1} (pre-cinacalcet application) after a 3 s step to −120 mV (Figures 6A and 6B). The time course of recovery of I_{S2} was described by a single exponential (T = 841 ± 72 ms; n = 10). In other words, cinacalcet slowed the time for 50% recovery from inactivation of I_{S2} by 1,180-fold (0.54 ms to 638 s; Figure 6B).

VGSC inactivation could arise from signals downstream of cinacalcet binding preferentially to specific channel states (Karoly et al., 2010) and thus be use dependent. We tested this hypothesis by examining the rate of VGSC current inhibition on duration of voltage step and the duty cycle of activation. VGSC currents were activated with depolarizing steps (5 or 30 ms) at rates of 0.2–5 Hz (Figure 6C). G-protein-mediated modulation of an ion channel is a multi-step process that has previously been shown to have complex kinetics (Yakubovich et al., 2005) that can be approximated by the function $f(t) = Ae^{-(t/\tau)^2} + B$ (equation 1). The kinetics of VGSC inhibition by cinacalcet were well described by this function, where t represents time, T the time constant of the inhibition, and A and B constants (Figure 6C). The modest change in time constant at different stimulation frequencies indicated little use-dependent inhibition at rates of 0.2–5 Hz stimulation (Figure 6D). In contrast, inhibition was slowed when we examined the action of cinacalcet at substantially lower rates of VGSC opening and closing (Figures 6C and 6E). VGSC currents were elicited by 30 ms depolarizing steps (−70 to −10 mV) at a frequency of 0.2 Hz but then paused immediately prior to cinacalcet (10 µM) application. After the first 200 s of cinacalcet application the voltage protocol was resumed, revealing that cinacalcet-mediated inhibition of VGSC

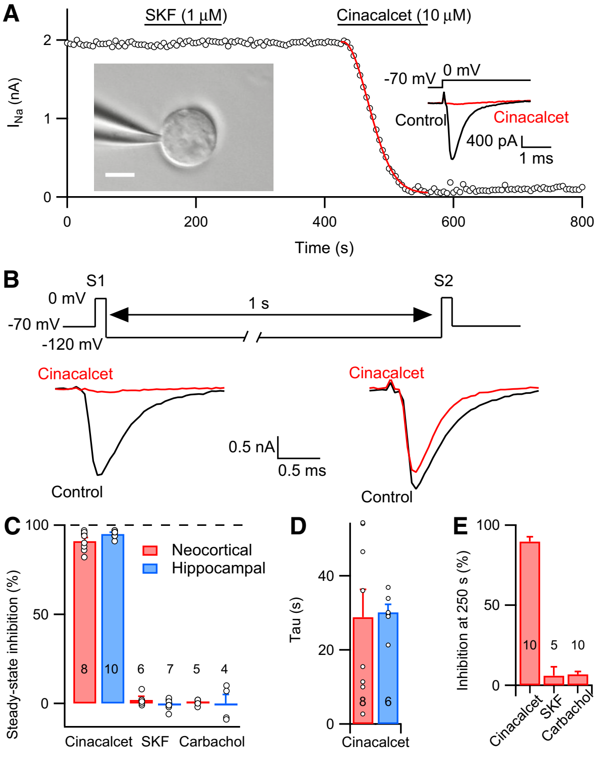

Figure 7. Cinacalcet Inhibits VGSC Current in Acutely Isolated Neurons and Is Reversed by Strong Hyperpolarization

(A) Diary plot of VGSC current in acutely isolated neocortical neuron during bath application of 10 μM cinacalcet and 1 μM D1-like dopamine receptor agonist SKF 81927. VGSC current was measured with 5 ms steps from −70 to 0 mV at a frequency of 0.2 Hz. Red line shows fit used on all datasets to calculate the time constant of the inhibition. Inset left: image of acutely isolated neocortical neuron during whole-cell patch-clamp recording. Scale bar indicates 15 μm. Inset right: representative traces show VGSC current in control conditions (ctrl, black) and at steady-state inhibition by cinacalcet (red).

(B) Representative traces from a double pulse protocol (S1, S2; 10 ms, 0 mV) used to elicit VGSC currents in control (black) or after complete block by 10 μM cinacalcet (red) (S1 and S2 separated by a 1 s recovery period at −120 mV).

(C) Bar graph showing steady-state inhibition of VGSC current produced by cinacalcet (10 μM), SKF 81927 (1 μM), or carbachol (20 μM) in acutely isolated neurons from the hippocampus (blue) or neocortex (red).

(D) Bar graph showing the time constant of the inhibition by cinacalcet (10 μM) from recordings of cells in the neocortex (red) and hippocampus (blue).

(E) Bar graph showing inhibition of VGSC current 250 s following drug exposure induced by cinacalcet (10 μM), SKF 81927 (1 μM), or carbachol (20 μM) in cultured neocortical neurons.

Error bars represent ± SEM.

currents was substantially smaller in the absence of the depolarizing steps (60% ± 7%; n = 7) than in control experiments (84% ± 4%; n = 11; p = 0.006; Figures 6C and 6E). These data indicate that cinacalcet-induced inhibition of VGSC is impaired at very low rates of channel activity and hence inhibition is use dependent.

Ca^{2+}-bound calmodulin (CaM) has been shown to bind to VGSCs and to shift VGSC inactivation (Tan et al., 2002; Yan et al., 2017). We hypothesized that increases in intracellular $[Ca^{2+}]$ ($[Ca^{2+}]_i$) might accelerate cinacalcet-mediated inhibition of VGSCs by facilitating inactivation. Using our standard protocol (Figure 1E), we found elevation of $[Ca^{2+}]_i$ by increasing Ca^{2+} entry via VACCs (increased bath $[Ca^{2+}]$; Figure S1) or by attenuating intracellular buffering (EDTA in pipette; data not shown) did not affect the action of cinacalcet on VGSC currents. Interestingly block of voltage-activated Ca^{2+} channels (VACCs) with the non-selective VACC blocker Cd^{2+} tended to slow the rate of VGSC current block by cinacalcet (Figure S1; p = 0.13). The data do not support the hypothesis that Ca^{2+}-bound CaM accelerates cinacalcet-mediated inhibition of VGSCs, but we cannot exclude the possibility that binding interactions such as these may contribute to use dependence.

Cinacalcet Inhibits VGSC Current in Acutely Isolated Neocortical Neurons

To rule out distortion of the kinetics of action of cinacalcet by voltage-clamp errors or long diffusion path lengths arising from neuronal processes, we examined VGSC currents in acutely isolated central neurons with short processes. VGSC currents were elicited with a 5 ms step from −70 to 0 mV. Just as in cultured neocortical neurons, VGSC currents in neurons isolated from acute neocortical and hippocampal slices were strongly and uniformly sensitive to cinacalcet (10 μM), inhibited by 91% ± 2% and 95% ± 1% in neocortical (n = 8) and hippocampal (n = 10) neurons, respectively (Figure 7C). The kinetics of cinacalcet inhibition of VGSC currents in these acutely isolated neurons were well described by equation 1 (Figure 7A). The rate of inhibition by cinacalcet was faster in the acutely isolated cells in comparison with cultured neocortical neurons (Figures 6D and 7D). Cinacalcet-mediated inhibition was also reversed by strong hyperpolarization in the acutely isolated neurons. A 1 s step to −120 mV almost completely relieved inhibition (Figure 7B). These data indicate that strong cinacalcet-mediated inhibition of VGSCs occurred in both acutely isolated and cultured cortical neurons.

G-protein-mediated inhibition of VGSC currents was described in a subgroup of neocortical and hippocampal neurons via activation of muscarinic acetylcholine M1 receptors (mAChR) and D1-like receptors (Cantrell et al., 1996, 1997). Although cinacalcet substantially inhibited VGSC currents in these recordings, the dopamine agonist SKF 81297 (1 μM) and mAChR M1 agonist carbachol (20 μM) had no effects on VGSC currents in acutely isolated neurons from the neocortex (Figures 7A and 7C) and hippocampus (Figure 7C) or in cultured neocortical neurons (Figure 7E), confirming that cinacalcet and these neurotransmitters act via distinct pathways.

DISCUSSION

VGSCs generate the upstroke of the action potential that has classically been described as a digital, all-or-none signal. We have described a pathway that is apparently ubiquitous in neocortical and hippocampal neurons and inhibits VGSCs in a graded manner by a G-protein-dependent mechanism. A number of features about this pathway are interesting. First, allosteric CaSR agonists stimulate this pathway. Second, this pathway appears independent of the CaSR because it is insensitive to external $[Ca^{2+}]$, occurs in $Casr^{-/-}$ mutants, and is also stimulated by allosteric CaSR antagonists. Third, this GTP-dependent

inhibition of VGSCs is not mediated by mGluR1, mGluR5, or $GABA_B$ receptors, which have strong structural homology with the CaSR. Fourth, this form of VGSC modulation is independent of PKA and PKC. Finally, the G-protein-mediated inhibition of VGSCs shifts steady-state inactivation of VGSCs and this can be reversed by prolonged hyperpolarization.

G-protein-mediated regulation of voltage-gated calcium and potassium channels has been a major area of scientific interest (Hille et al., 2014; Holz et al., 1986). In contrast, G-protein regulation of VGSCs has received substantially less attention. Earlier studies showed that VGSC currents in the neocortex and hippocampus were reduced by ~20%–40% through mAChR M1, D1-like receptor, mGluR1, and serotonin 5-$HT_{2a/c}$ receptor activation (Cantrell et al., 1996, 1997; Carlier et al., 2006; Carr et al., 2002). In contrast, our findings show near complete inhibition of VGSCs is possible in a large majority of cortical neurons, and this points to the existence of a signaling pathway that could substantially regulate neuronal activity in the cortex. The effectiveness of GDPβS to block the action of all four tested ACMs on VGSC currents and GTPγS to accelerate the rate of decrease of basal VGSC currents strongly indicate the involvement of G-proteins in the pathway (Figure 3). These established tools competitively inhibit endogenous ligands interacting with the G-protein nucleotide-binding pocket (Oldham et al., 2007; Suh et al., 2004). Non-hydrolyzable GDPβS reduces G-protein activation by GTP despite GPCR activation, whereas GTPγS will enhance G-protein signaling because it attenuates endogenous nucleotide hydrolysis that terminates G-protein activity (Oldham et al., 2007). The likelihood of GDPβS acting via unidentified G-protein-independent pathways seemed low because we excluded direct chemical modification of cinacalcet, off-target effects of the terminal sulfur atom, and reduced sensitivity of VGSC to direct blockers as causes for GDPβS block of cinacalcet-mediated VGSC inhibition (Figure 3). Another possibility is that cinacalcet blocks VGSCs by directly binding to and stabilizing a slow inactivated state of the channel and that GDPβS modulates the high-affinity state to prevent cinacalcet binding. The inability of GDPβS to affect VGSC block by phenytoin or carbamazepine makes this mechanism less likely but does not rule it out. On balance our data point to VGSC inhibition by cinacalcet being mediated by G-proteins.

A number of questions remain about the mechanism of inhibition of VGSCs by ACMs. What is the identity of the GPCR that mediates the effects of ACMs on VGSCs? We found that cinacalcet-mediated inhibition was independent of the CaSR and other class C GPRCs: mGluR1, mGluR5, and the $GABA_B$ receptor (Figures 4B and 4D). Cinacalcet-mediated inhibition of VGSCs was also distinguished from other GPCR-mediated pathways (Cantrell et al., 1996, 1997; Carlier et al., 2006; Carr et al., 2002) by its resistance to PKA and PKC inhibition (Figure 4H). Stimulation of mAChR receptors and D1-like receptors did not reduce VGSC currents in cinacalcet-sensitive neurons, indicating further separation between the mechanisms underlying cinacalcet-mediated and other forms of G-protein-mediated inhibition of VGSCs (Figures 7A, 7C, and 7E). The candidate molecular targets for GPCR-activated PKA and PKC inhibition of VGSCs include serine residues on the α subunit of the channel, but this has not been fully resolved (Rossie et al., 1987; Smith and Goldin, 1996). VGSCs and VACCs share a number of properties (Ben-Johny et al., 2015), and by analogy with VACCs, where G-protein interactions appear complex, there may be several sites at which VGSCs are targeted by G-proteins (Proft and Weiss, 2015). Interestingly, although G-protein regulation of VGSCs is reversed by strong hyperpolarization (Figures 6A, 6B, and 7B) G-protein-mediated inhibition of VACCs is reversed by strong depolarization (Bean, 1989; Namkung et al., 1998). One possible mechanism for cinacalcet-induced inhibition of VGSCs is the direct action of the Gβγ dimer of the G-protein complex on these channels. This type of G-protein-to-ion channel interaction has been observed with both potassium and calcium channels (Ikeda, 1996; Navarro et al., 1996). Interestingly, $G\beta_2\gamma_3$ has been shown to interact with $Na_V1.2$ at the C terminus, and this interaction increases persistent VGSC current in proportion to transient VGSC current in tsA-201 cells (Mantegazza et al., 2005). Future experiments will address the identity of the major players responsible for inhibition of VGSCs by ACMs.

Acting indirectly, cinacalcet promoted VGSC inactivation and so decreased VGSC availability at −70 mV. This provides the mechanism of VGSC inhibition by cinacalcet (Figure 5E) and reassuringly is similar to how other G-protein-mediated forms of VGSC inhibition occurred (Carr et al., 2002). Prolonged hyperpolarization reversed cinacalcet-mediated VGSC modulation, indicating that the partial reversibility (Figure 1) did not indicate VGSC loss or rundown. Instead, the slow recovery from inactivation following strong hyperpolarization (Figure 6B) may be due to the promotion of slow VGSC inactivation or to slow dissociation of blocking molecules from the fast inactivation state (Karoly et al., 2010). Certainly the near complete relief of ACM-induced inhibition of VGSCs by the G-protein signaling blocker GDPβS suggests that this effect is due to an indirect action of cinacalcet on VGSCs, in contrast to the use-dependent pore blockers (Kuo and Bean, 1994). Cinacalcet-mediated inhibition of VGSCs was significantly reduced at very low frequencies of VGSC activation (Figures 6C and 6D), implying that low rates of VGSC cycling will attenuate the effectiveness of the G-protein-dependent pathway or conversely that the pathway will become more influential when neuronal excitability is increased. Elevating $[Ca^{2+}]_i$ by increasing Ca^{2+} entry via VACCs and by attenuating intracellular buffering or decreasing $[Ca^{2+}]_i$ by reducing Ca^{2+} entry via VACCs did not affect cinacalcet-mediated inhibition of VGSC currents, indicating no substantial Ca^{2+}-binding protein CaM-VGSC interaction underlying this pathway.

What are the other functional implications for this pathway? Multiple lines of evidence indicate that VGSC density and gating characteristics are important in shaping action potentials within a specific neuron (Bean, 2007; Lewis and Raman, 2014). VGSC current inhibition that relies on slow inactivation has been shown to reduce a neuron's ability to sustain trains of spikes (Carr et al., 2003). We predict that the strong, slow inhibition of VGSC by ACMs should have similar effects. In addition to modulating general cellular excitability, the pathway may have other important actions. Inhibition of VGSCs in a branching axon provides a mechanism by which failures in synaptic transmission could be explained (Figure 1B). Regulation of action potential propagation throughout the axonal arbor has been proposed as an important form of synaptic plasticity (Debanne, 2004). One such example is

in the nucleus of the solitary tract where a fraction of synapses respond to arginine vasopressin (AVP) by switching from a release probability of 0.9 at ~20 release sites in the same axon to complete failure of transmission (Bailey et al., 2006), possibly because of failure of propagation at an axonal branchpoint. Identification of the receptor by which cinacalcet inhibits VGSC current may allow us to determine if such a mechanism contributes to synaptic plasticity.

Cinacalcet has been used to treat forms of hyperparathyroidism in an attempt to reduce the complications of elevated serum calcium levels (Nemeth and Goodman, 2016). Despite reducing parathyroid hormone (PTH) levels, cinacalcet did not reduce mortality (EVOLVE Trial Investigators et al., 2012). Could harmful off-target effects in neurons explain cinacalcet's apparent lack of efficacy? It may seem unlikely given that at clinical doses cinacalcet serum levels are ~50 nM (Padhi and Harris, 2009), so that only 2% of the VGSCs would be blocked (Figure 1E). However, calculations for phenytoin suggest that similarly small fractions of VGSCs are blocked by clinically effective doses (Kane et al., 2013). Moreover, cinacalcet's high volume of distribution and high partition coefficient indicate that higher brain concentrations due to accumulation are likely. Consequently, we cannot dismiss the possibility that clinically important off-target effects of cinacalcet may arise from VGSC block. CaSR modulators that are not lipophilic and less likely to cross the blood-brain barrier are being synthesized and tested clinically (Martin et al., 2014), indicating that cinacalcet actions in the brain may be important.

In conclusion, we have shown that a broad range of GPCR modulators block VGSC currents in a GTP-dependent fashion. The strength of block and reversal by hyperpolarization confirm that this mechanism is positioned to regulate neuronal excitability under a range of physiological and pathological conditions.

EXPERIMENTAL PROCEDURES

Neuronal Cell Culture

Neocortical neurons were isolated from postnatal day 1–2 mouse pups of either sex, as described previously (Phillips et al., 2008). All animal procedures were approved by the VA Portland Health Care System Institutional Animal Care and Use Committee in accordance with the U.S. Public Health Service Policy on Humane Care and Use of Laboratory Animals and the NIH Guide for the Care and Use of Laboratory Animals. Animals were decapitated following general anesthetic with isoflurane, and then the cerebral cortices were removed. Cortices were incubated in trypsin and DNase and then dissociated with a heat-polished pipette. Dissociated cells were cultured in MEM plus 5% fetal bovine serum (FBS) on glass coverslips. Cytosine arabinoside (4 μM) was added 48 hr after plating to limit glial division. Cells were used between 1 and 12 days in culture. Homozygous lox CaSR, nestin-cre negative females and positive males were mated to produce conditional cre $Casr^{-/-}$ mutants (Chang et al., 2008). DNA extraction was performed using the Hot Shot Technique (Truett et al., 2000) with a 1–2 hr boil. Primers used for cre PCR were Nes-Cre 1: GCAAAACAGGCTCTAGCGTTCG; Nes-Cre 2: CTGTTTCACTATCCAGGTTACGG; run on a 1% agarose gel. Primers for lox PCR were P3U: TGTGACGGAAAACATACTGC; Lox R: GCGTTTTTAGAGG GAAGCAG; run on a 1.5% agarose gel.

Acute Isolated Neurons

Mice postnatal day 11–19 were decapitated under anesthesia, and brain was rapidly dissected and placed in chilled, oxygenated (4°C, 95% O_2, 5% CO_2) choline chloride-based artificial cerebrospinal fluid (ACSF), and horizontal or coronal slices (400 μm thick) were cut with a vibratome (Leica VT 1200S). Slices were incubated in standard ACSF for 1 hr and then treated for 30–40 min with 0.5 mg/mL protease type XIV (Sigma-Aldrich) in Tyrode's solution (below) containing only 100 μM of $CaCl_2$. After enzyme treatment, slices were rinsed with standard ACSF and mechanically dissociated using glass pipettes of decreasing size. Cells were used <1 hr after dissociation.

Electrophysiological Recordings

Cells were visualized with a Nikon Diaphot, Leica DM IRB inverted microscope, or Scientifica SliceScope. Whole-cell voltage- and current-clamp recordings were made from cultured neocortical neurons using a HEKA EPC10 USB amplifier or Axoclamp 200B. Except where stated in the text, extracellular Tyrode's solution contained 150 mM NaCl, 4 mM KCl, 10 mM HEPES, 10 mM glucose, 1.1 mM $MgCl_2$, and 1.1 mM $CaCl_2$ (pH 7.35) with NaOH. Extracellular choline chloride-based ACSF (ChACSF) contained 122 mM choline chloride, 2.5 mM KCl, 1.25 mM NaH_2PO_4, 25 mM $NaHCO_3$, 8 mM glucose, 0.8 mM $CaCl_2$, and 4 mM $MgCl_2$. Extracellular standard ACSF contained 129 mM NaCl, 3.3 mM KCl, 25 mM $NaHCO_3$, 5 mM glucose, 0.4 mM Na_2HPO_4, 0.4 mM KH_2PO_4, 1 mM $MgCl_2$, and 1.5 mM $CaCl_2$. VGSC current recordings were made using a cesium methane-sulfonate intracellular solution containing 113 mM $CsMeSO_3$, 1.8 mM EGTA, 10 mM HEPES, 4 mM $MgCl_2$, 0.2 mM $CaCl_2$, 4 mM NaATP, 0.3 mM NaGTP, and 14 mM creatine phosphate (pH 7.2) with TEA hydroxide. In some experiments, GTP was replaced with 2 mM GDPβS (Figure 3), 2 mM ADPβS (Figures 3E and 3F), or 0.5 mM GTPγS (Figure 3G). IPSCs (Figure 1B) were recorded using a KCl-rich intracellular solution containing 118 mM KCl, 1 mM EGTA, 10 mM HEPES, 4 mM $MgCl_2$, 1 mM $CaCl_2$, 4 mM NaATP, 0.3 mM NaGTP, 14 mM creatinine phosphate, and 1 mM QX-314 (pH 7.2) with KOH. To pharmacologically isolate IPSCs, 10 μM CNQX was added to the bath. IPSCs were completely blocked by 40 μM gabazine or 10 μM bicuculline, indicating that they were mediated by GABA. Recordings of action potentials (Figure 1C) were made using a potassium gluconate-rich intracellular solution containing 135 mM K-gluconate, 10 mM HEPES, 4 mM $MgCl_2$, 4 mM NaATP, 0.3 mM NaGTP, and 10 mM creatinine phosphate (pH 7.2) with KOH. To isolate action potentials, 40 μM CNQX, 80 μM APV, and 40 μM gabazine were added to the bath. Electrodes used for recording had resistances of 2–4 MΩ. Voltages indicated have been corrected for liquid junction potentials. All experiments were performed at room temperature (20°C–24°C).

Data Acquisition and Analysis

Whole-cell voltage- and current-clamp recordings were filtered at 3–5 kHz using a Bessel filter and sampled at 100 kHz. Leak current was subtracted online using a –p/n protocol. Rs was compensated by 60%–90%. Analysis was performed using Igor Pro (Wavemetrics, Lake Oswego, OR). Unless otherwise stated, recordings were only included if the rate of baseline rundown was <10% over 100 s. Data values are reported as mean ± SEM. Statistical significance was determined using Student's t test, two-tailed (Microsoft Excel), unless otherwise noted. The action of nucleotide on the rate of VGSC current rundown was evaluated using a two-way RM ANOVA (GraphPad Prism version 6). ANOVA is reported in Table S1.

Solution Application

Solutions were gravity-fed through a glass capillary (1.2 mm outer diameter) placed ~1 mm from the patch pipette tip. Most reagents were obtained from Sigma-Aldrich (Darmstadt, Germany). NPS 2143, PKI 19-36, staurosporine, saclofen, SKF 81297, carbachol, and CHPG were supplied by Tocris (Bristol, United Kingdom). PKI 6-22, JNJ 16259685, and baclofen were supplied by Santa Cruz Biotechnology (Dallas, United States). Pertussis toxin was supplied by Millipore Sigma (Burlington, Massachusetts). Cinacalcet was supplied by Toronto Research Chemicals (Toronto, Canada) and TTX by Alomone (Jerusalem, Israel). Phenytoin, carbamazepine, and CHPG were dissolved in DMSO (final concentration 0.125%). NPS 2143, calhex, MPEP, DMeOB, staurosporine, and chelerythrine chloride were dissolved in DMSO (final concentration ≤ 0.03%). JNJ 16259685 was dissolved in ethanol (final concentration 0.05%). Appropriate vehicle controls were performed for all experiments.

SUPPLEMENTAL INFORMATION

Supplemental Information includes Supplemental Experimental Procedures, one figure, and one table and can be found with this article online at https://doi.org/10.1016/j.celrep.2018.04.109.

ACKNOWLEDGMENTS

The Casr$^{-/-}$ mice were a kind gift of Dr. Wenhan Chang, University of California, San Francisco (UCSF) and San Francisco Veterans Affairs Medical Center. We are grateful to Dr. Courtney Williams for performing the experiments on synaptic transmission and to Ms. Briana Knight for help with cell culture. We thank the Smith lab members and Drs. Henrique von Gersdorff, Laurence Trussell, and John Williams for productive discussion. This work was supported by grants awarded by the National Institute of General Medical Sciences (NIGMS) (R01 GM097433) and the U.S. Department of Veterans Affairs (BX002547) to S.M.S. In addition, G.B.M. was supported by Achievement Rewards for College Scientists (ARCS), the National Heart, Lung, and Blood Institute (NHLBI) (T32HL083808), and the National Institute of Neurological Disorders and Stroke (NINDS) (F31NS095463). The contents do not represent the views of the U.S. Department of Veterans Affairs or the United States Government.

AUTHOR CONTRIBUTIONS

G.B.M. and S.M.S. designed the study and wrote the manuscript. G.B.M., S.M.S., and T.T. conducted the experiments and analyzed the data.

DECLARATION OF INTERESTS

The authors declare no competing interests.

Received: October 12, 2017
Revised: March 26, 2018
Accepted: April 25, 2018
Published: May 29, 2018

REFERENCES

Aman, T.K., Grieco-Calub, T.M., Chen, C., Rusconi, R., Slat, E.A., Isom, L.L., and Raman, I.M. (2009). Regulation of persistent Na current by interactions between beta subunits of voltage-gated Na channels. J. Neurosci. *29*, 2027–2042.

Bailey, T.W., Jin, Y.H., Doyle, M.W., Smith, S.M., and Andresen, M.C. (2006). Vasopressin inhibits glutamate release via two distinct modes in the brainstem. J. Neurosci. *26*, 6131–6142.

Bean, B.P. (1989). Neurotransmitter inhibition of neuronal calcium currents by changes in channel voltage dependence. Nature *340*, 153–156.

Bean, B.P. (2007). The action potential in mammalian central neurons. Nat. Rev. Neurosci. *8*, 451–465.

Ben-Johny, M., Dick, I.E., Sang, L., Limpitikul, W.B., Kang, P.W., Niu, J., Banerjee, R., Yang, W., Babich, J.S., Issa, J.B., et al. (2015). Towards a unified theory of calmodulin regulation (calmodulation) of voltage-gated calcium and sodium channels. Curr. Mol. Pharmacol. *8*, 188–205.

Cantrell, A.R., Ma, J.Y., Scheuer, T., and Catterall, W.A. (1996). Muscarinic modulation of sodium current by activation of protein kinase C in rat hippocampal neurons. Neuron *16*, 1019–1026.

Cantrell, A.R., Smith, R.D., Goldin, A.L., Scheuer, T., and Catterall, W.A. (1997). Dopaminergic modulation of sodium current in hippocampal neurons via cAMP-dependent phosphorylation of specific sites in the sodium channel alpha subunit. J. Neurosci. *17*, 7330–7338.

Cantrell, A.R., Scheuer, T., and Catterall, W.A. (1999). Voltage-dependent neuromodulation of Na+ channels by D1-like dopamine receptors in rat hippocampal neurons. J. Neurosci. *19*, 5301–5310.

Carlier, E., Sourdet, V., Boudkkazi, S., Déglise, P., Ankri, N., Fronzaroli-Molinieres, L., and Debanne, D. (2006). Metabotropic glutamate receptor subtype 1 regulates sodium currents in rat neocortical pyramidal neurons. J. Physiol. *577*, 141–154.

Carr, D.B., Cooper, D.C., Ulrich, S.L., Spruston, N., and Surmeier, D.J. (2002). Serotonin receptor activation inhibits sodium current and dendritic excitability in prefrontal cortex via a protein kinase C-dependent mechanism. J. Neurosci. *22*, 6846–6855.

Carr, D.B., Day, M., Cantrell, A.R., Held, J., Scheuer, T., Catterall, W.A., and Surmeier, D.J. (2003). Transmitter modulation of slow, activity-dependent alterations in sodium channel availability endows neurons with a novel form of cellular plasticity. Neuron *39*, 793–806.

Chang, W., Tu, C., Chen, T.-H., Bikle, D., and Shoback, D. (2008). The extracellular calcium-sensing receptor (CaSR) is a critical modulator of skeletal development. Sci. Signal. *1*, ra1.

Chen, W., Bergsman, J.B., Wang, X., Gilkey, G., Pierpoint, C.R., Daniel, E.A., Awumey, E.M., Dauban, P., Dodd, R.H., Ruat, M., and Smith, S.M. (2010). Presynaptic external calcium signaling involves the calcium-sensing receptor in neocortical nerve terminals. PLoS ONE *5*, e8563.

EVOLVE Trial Investigators; Chertow, G.M., Block, G.A., Correa-Rotter, R., Drüeke, T.B., Floege, J., Goodman, W.G., Herzog, C.A., Kubo, Y., London, G.M., Mahaffey, K.W., et al. (2012). Effect of cinacalcet on cardiovascular disease in patients undergoing dialysis. N. Engl. J. Med. *367*, 2482–2494.

Cusack, N.J., and Hourani, S.M. (1981). Partial agonist behaviour of adenosine 5′-O-(2-thiodiphosphate) on human platelets. Br. J. Pharmacol. *73*, 405–408.

Debanne, D. (2004). Information processing in the axon. Nat. Rev. Neurosci. *5*, 304–316.

Eckstein, F., Cassel, D., Levkovitz, H., Lowe, M., and Selinger, Z. (1979). Guanosine 5′-O-(2-thiodiphosphate). An inhibitor of adenylate cyclase stimulation by guanine nucleotides and fluoride ions. J. Biol. Chem. *254*, 9829–9834.

Hille, B., Dickson, E., Kruse, M., and Falkenburger, B. (2014). Dynamic metabolic control of an ion channel. Prog. Mol. Biol. Transl. Sci. *123*, 219–247.

Holz, G.G., 4th, Rane, S.G., and Dunlap, K. (1986). GTP-binding proteins mediate transmitter inhibition of voltage-dependent calcium channels. Nature *319*, 670–672.

Huang, H., and Trussell, L.O. (2008). Control of presynaptic function by a persistent Na(+) current. Neuron *60*, 975–979.

Ikeda, S.R. (1996). Voltage-dependent modulation of N-type calcium channels by G-protein beta gamma subunits. Nature *380*, 255–258.

Jo, S., and Bean, B.P. (2011). Inhibition of neuronal voltage-gated sodium channels by brilliant blue G. Mol. Pharmacol. *80*, 247–257.

Kane, S.P., Bress, A.P., and Tesoro, E.P. (2013). Characterization of unbound phenytoin concentrations in neurointensive care unit patients using a revised Winter-Tozer equation. Ann. Pharmacother. *47*, 628–636.

Kaplan, D.I., Isom, L.L., and Petrou, S. (2016). Role of sodium channels in epilepsy. Cold Spring Harb. Perspect. Med. *6*, 6.

Karoly, R., Lenkey, N., Juhasz, A.O., Vizi, E.S., and Mike, A. (2010). Fast- or slow-inactivated state preference of Na+ channel inhibitors: a simulation and experimental study. PLoS Comput. Biol. *6*, e1000818.

Kuo, C.C., and Bean, B.P. (1994). Slow binding of phenytoin to inactivated sodium channels in rat hippocampal neurons. Mol. Pharmacol. *46*, 716–725.

Lambright, D.G., Noel, J.P., Hamm, H.E., and Sigler, P.B. (1994). Structural determinants for activation of the alpha-subunit of a heterotrimeric G protein. Nature *369*, 621–628.

Leach, K., Conigrave, A.D., Sexton, P.M., and Christopoulos, A. (2015). Towards tissue-specific pharmacology: insights from the calcium-sensing receptor as a paradigm for GPCR (patho)physiological bias. Trends Pharmacol. Sci. *36*, 215–225.

Leão, R.M., Kushmerick, C., Pinaud, R., Renden, R., Li, G.L., Taschenberger, H., Spirou, G., Levinson, S.R., and von Gersdorff, H. (2005). Presynaptic Na+ channels: locus, development, and recovery from inactivation at a high-fidelity synapse. J. Neurosci. *25*, 3724–3738.

Lewis, A.H., and Raman, I.M. (2014). Resurgent current of voltage-gated Na(+) channels. J. Physiol. *592*, 4825–4838.

Mantegazza, M., Yu, F.H., Powell, A.J., Clare, J.J., Catterall, W.A., and Scheuer, T. (2005). Molecular determinants for modulation of persistent sodium current by G-protein betagamma subunits. J. Neurosci. *25*, 3341–3349.

Martin, K.J., Bell, G., Pickthorn, K., Huang, S., Vick, A., Hodsman, P., and Peacock, M. (2014). Velcalcetide (AMG 416), a novel peptide agonist of the calcium-sensing receptor, reduces serum parathyroid hormone and FGF23 levels in healthy male subjects. Nephrol. Dial. Transplant. *29*, 385–392.

Namkung, Y., Smith, S.M., Lee, S.B., Skrypnyk, N.V., Kim, H.L., Chin, H., Scheller, R.H., Tsien, R.W., and Shin, H.S. (1998). Targeted disruption of the Ca2+ channel beta3 subunit reduces N- and L-type Ca2+ channel activity and alters the voltage-dependent activation of P/Q-type Ca2+ channels in neurons. Proc. Natl. Acad. Sci. U S A *95*, 12010–12015.

Navarro, B., Kennedy, M.E., Velimirovíc, B., Bhat, D., Peterson, A.S., and Clapham, D.E. (1996). Nonselective and G betagamma-insensitive weaver K+ channels. Science *272*, 1950–1953.

Nemeth, E.F., and Goodman, W.G. (2016). Calcimimetic and calcilytic drugs: feats, flops, and futures. Calcif. Tissue Int. *98*, 341–358.

Neves, S.R., Ram, P.T., and Iyengar, R. (2002). G protein pathways. Science *296*, 1636–1639.

Oldham, W.M., and Hamm, H.E. (2008). Heterotrimeric G protein activation by G-protein-coupled receptors. Nat. Rev. Mol. Cell Biol. *9*, 60–71.

Oldham, W.M., Van Eps, N., Preininger, A.M., Hubbell, W.L., and Hamm, H.E. (2007). Mapping allosteric connections from the receptor to the nucleotide-binding pocket of heterotrimeric G proteins. Proc. Natl. Acad. Sci. U S A *104*, 7927–7932.

Padhi, D., and Harris, R. (2009). Clinical pharmacokinetic and pharmacodynamic profile of cinacalcet hydrochloride. Clin. Pharmacokinet. *48*, 303–311.

Phillips, C.G., Harnett, M.T., Chen, W., and Smith, S.M. (2008). Calcium-sensing receptor activation depresses synaptic transmission. J. Neurosci. *28*, 12062–12070.

Pitt, G.S., and Lee, S.Y. (2016). Current view on regulation of voltage-gated sodium channels by calcium and auxiliary proteins. Protein Sci. *25*, 1573–1584.

Proft, J., and Weiss, N. (2015). G protein regulation of neuronal calcium channels: back to the future. Mol. Pharmacol. *87*, 890–906.

Raman, I.M., and Bean, B.P. (1997). Resurgent sodium current and action potential formation in dissociated cerebellar Purkinje neurons. J. Neurosci. *17*, 4517–4526.

Rogawski, M.A., Löscher, W., and Rho, J.M. (2016). Mechanisms of Action of Antiseizure Drugs and the Ketogenic Diet. Cold Spring Harb. Perspect. Med. *6*, 6.

Rossie, S., Gordon, D., and Catterall, W.A. (1987). Identification of an intracellular domain of the sodium channel having multiple cAMP-dependent phosphorylation sites. J. Biol. Chem. *262*, 17530–17535.

Seifert, R., and Wenzel-Seifert, K. (2002). Constitutive activity of G-protein-coupled receptors: cause of disease and common property of wild-type receptors. Naunyn Schmiedebergs Arch. Pharmacol. *366*, 381–416.

Smith, R.D., and Goldin, A.L. (1996). Phosphorylation of brain sodium channels in the I–II linker modulates channel function in Xenopus oocytes. J. Neurosci. *16*, 1965–1974.

Smith, S.M., Chen, W., Vyleta, N.P., Williams, C., Lee, C.H., Phillips, C., and Andresen, M.C. (2012). Calcium regulation of spontaneous and asynchronous neurotransmitter release. Cell Calcium *52*, 226–233.

Suh, B.C., Horowitz, L.F., Hirdes, W., Mackie, K., and Hille, B. (2004). Regulation of KCNQ2/KCNQ3 current by G protein cycling: the kinetics of receptor-mediated signaling by Gq. J. Gen. Physiol. *123*, 663–683.

Tan, H.L., Kupershmidt, S., Zhang, R., Stepanovic, S., Roden, D.M., Wilde, A.A., Anderson, M.E., and Balser, J.R. (2002). A calcium sensor in the sodium channel modulates cardiac excitability. Nature *415*, 442–447.

Truett, G.E., Heeger, P., Mynatt, R.L., Truett, A.A., Walker, J.A., and Warman, M.L. (2000). Preparation of PCR-quality mouse genomic DNA with hot sodium hydroxide and tris (HotSHOT). Biotechniques *29*, 52, 54.

Ui, M. (1984). Islet-activating protein, pertussis toxin: a probe for functions of the inhibitory guanine nucleotide regulatory component of adenylate cyclase. Trends Pharmacol. Sci. *5*, 277–279.

Urwyler, S. (2011). Allosteric modulation of family C G-protein-coupled receptors: from molecular insights to therapeutic perspectives. Pharmacol. Rev. *63*, 59–126.

Yakubovich, D., Rishal, I., and Dascal, N. (2005). Kinetic modeling of Na(+)-induced, Gbetagamma-dependent activation of G protein-gated K(+) channels. J. Mol. Neurosci. *25*, 7–19.

Yan, H., Wang, C., Marx, S.O., and Pitt, G.S. (2017). Calmodulin limits pathogenic Na+ channel persistent current. J. Gen. Physiol. *149*, 277–293.

Zeng, Z., Hill-Yardin, E.L., Williams, D., O'Brien, T., Serelis, A., and French, C.R. (2016). Effect of phenytoin on sodium conductances in rat hippocampal CA1 pyramidal neurons. J. Neurophysiol. *116*, 1924–1936.

Cell Reports, Volume *23*

Supplemental Information

Strong G-Protein-Mediated Inhibition of Sodium Channels

Glynis B. Mattheisen, Timur Tsintsadze, and Stephen M. Smith

SUPPLEMENTAL FIGURES AND LEGENDS

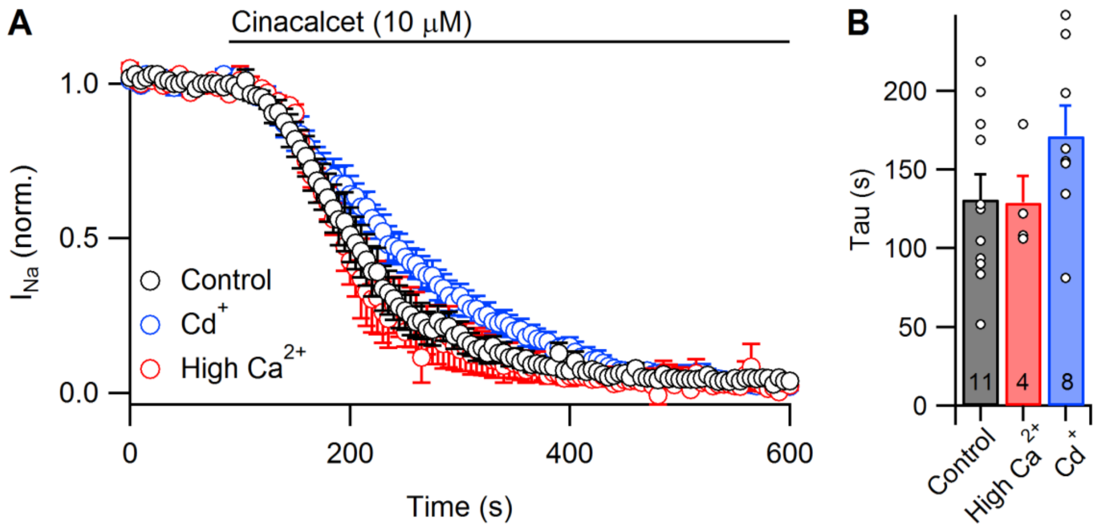

Figure S1. Cinacalcet inhibition is independent of extracellular calcium. Related to Figure 2. (A) Diary plot of VGSC current in cultured neocortical neurons during bath application of 10 µM cinacalcet in control condition (black; n =11), in the presence of 50 µM cadmium (Cd^{+}; n = 8; blue) to block VACCs, and in the presence of 10 mM extracellular calcium (High Ca^{2+}; n = 4; red). VGSC current was measured with 5 ms steps from -70 mV to 0 mV at 0.2 Hz. (B) Bar graph showing the time constant of the inhibition (Tau) by cinacalcet (10 µM) in control conditions (black), in the presence of 50 µM cadmium (Cd^{+}; blue), and in the presence of 10 mM extracellular calcium (High Ca^{2+}; red). Error bars show mean value ± SEM.

Table S1. **GTPγS accelerated VGSC rundown compared with GTP and GDPβS.** Related to Figure 3H.

ANOVA table	SS	DF	MS	F (DFn, DFd)	P value
**Interaction**	1.162	68	0.01710	F (68, 1768) = 2.127	P < 0.0001
**Time**	13.25	34	0.3898	F (34, 1768) = 48.50	P < 0.0001
**nucleotide**	5.993	2	2.997	F (2, 52) = 3.708	P = 0.0312
**Subjects (matching)**	42.03	52	0.8082	F (52, 1768) = 100.6	P < 0.0001
**Residual**	14.21	1768	0.008038		

SUPPLEMENTAL EXPERIMENTAL PROCEDURES

Resource Table

REAGENT or RESOURCE	SOURCE	IDENTIFIER
**Chemicals, Peptides, and Recombinant Proteins**		
Cinacalcet	Toronto Research Chemicals	Cat#C441803
Calindol	Sigma-Aldrich	CAS: 729610-18-8
NPS 2143	Tocris Bioscience	Cat#3626
Calhex	Sigma-Aldrich	CAS: 652973-93-8
**Experimental Models: Organisms/Strains**		
Mus musculus: C57/BL6J & 129S4: $^{nes\text{-}cre}CaSR^{\Delta flox/\Delta flox}$	Laboratory of Dr. Wenhan Chang	Chang, *et al.,* 2008
Mus musculus: C57BL/6J x 129X1: A^W/A^W	The Jackson Laboratory	RRID:MGI:5652742
